# Recommendations to Enable Broader Use of Real‐World Evidence to Inform Decision‐Making Throughout Pharmacovigilance Signal Management

**DOI:** 10.1002/pds.70231

**Published:** 2025-10-09

**Authors:** G. Niklas Norén, Katherine Donegan, Monica A. Muñoz, Thamir M. Alshammari, Nicole Pratt, Gianmario Candore, Daniel Morales, Peter Rijnbeek, Andrew Bate, Rodrigo Postigo, Sengwee Toh, Gianluca Trifirò, Montse Soriano Gabarro, Alison Cave, Patrick B. Ryan

**Affiliations:** ^1^ Uppsala Monitoring Centre Uppsala Sweden; ^2^ Medicines and Healthcare Products Regulatory Agency London UK; ^3^ US Food and Drug Administration Silver Spring Maryland USA; ^4^ Jazan University Jazan Saudi Arabia; ^5^ University of South Australia Adelaide Australia; ^6^ Bayer AG Berlin Germany; ^7^ European Medicines Agency Amsterdam the Netherlands; ^8^ Erasmus University Medical Center Rotterdam the Netherlands; ^9^ GSK London UK; ^10^ Harvard Medical School Boston Massachusetts USA; ^11^ Harvard Pilgrim Health Care Institute Boston Massachusetts USA; ^12^ University of Verona Italy; ^13^ Janssen Research and Development Titusville New Jersey USA

**Keywords:** decision‐making, pharmacovigilance, real‐world data, real‐world evidence, regulatory science, signal management

## Abstract

**Introduction:**

Despite substantial investments in analytical infrastructure and scientific research related to the development and analysis of real‐world evidence in support of signal management, the impact on routine pharmacovigilance activities has been limited. Most organizations still rely largely on analyses of individual case reports and pre‐existing evidence – especially during signal detection and validation.

**Objective:**

This paper presents a set of recommendations for efforts to enable broader use of real‐world evidence throughout pharmacovigilance signal management, in the future.

**Outcome:**

The recommendations regard streamlined data access, data harmonization and use of reproducible analytical workflows to enable rapid and robust evidence generation. They emphasize the need for cross‐disciplinary collaboration and for organizational adaptations to ensure adequate competence and supporting processes, including principles for how to integrate new types of evidence in decision‐making. The execution of pilot studies under realistic conditions and the dissemination of their findings are highlighted as important steps toward defining the proposed change and driving progress in this area. This manuscript is endorsed by the International Society for Pharmacoepidemiology (ISPE).

**Summary**

- Substantial investments have been made to support the development and analysis of real‐world evidence for regulatory decision‐making
- Even so, most pharmacovigilance organizations rely primarily on individual case reports and preexisting evidence during signal management
- Streamlined access to fit‐for‐purpose data, data harmonization, and the use of reproducible analytical workflows are identified as enablers of rapid and robust evidence generation using real‐world data
- Impact on pharmacovigilance decision‐making may depend on cross‐disciplinary collaboration and the establishment of principles for evidence integration
- The execution of pilot studies and dissemination of their findings can help drive progress

## 1 Background

Pharmacovigilance is the science and activities relating to the detection, assessment, understanding, and prevention of adverse effects or any other problem related to medicinal products. Medicinal products approved for regular clinical use must be continually monitored for new information that may alter their benefit–risk balance overall and/or in different settings and populations. To this end, regulatory authorities, pharmaceutical companies, and other stakeholders analyze an array of data sources to detect information that may suggest previously unknown risks of adverse effects or new information about known adverse effects. This is referred to as *signal detection*. The broader process of detecting, prioritizing, and evaluating pharmacovigilance signals that may represent a risk is referred to as *signal management* [1].

Historically, signal management has relied extensively on individual case safety reports which describe adverse events in association with the use of medical products and interventions. Such reports may be submitted by patients and health professionals and are systematically collected by regulatory authorities and pharmaceutical companies. Analysis of these reports informs most regulatory decisions related to safety signals concerning marketed medicinal products [2, 3, 4]. Yet, the analysis of routinely collected health data such as electronic medical records and claims may play a role in signal detection for specific adverse drug reactions [5].

It has been argued that the increased availability and heterogeneity of routinely collected data relating to a patient's health status or the delivery of health care and more powerful analytical capabilities may enable epidemiological evidence generated from such real‐world data (RWD) to play a more relevant role in pharmacovigilance signal management [6, 7, 8, 9]. A recent position statement by the Real‐World Evidence and Big Data Special Interest Group of the International Society of Pharmacovigilance (ISoP) further elaborates the possible complementarity and interplay of individual case reports and RWD in signal management [10]. Large collaborative research networks that harmonize data capture using common data models (CDM) and utilize reproducible analytical workflows to enable rapid development of real‐world evidence (RWE) have been established and could improve the process of signal management at different stages, even for less common medicinal products and adverse events [8, 11, 12]. Substantial investments have been made in developing analytical infrastructure and carrying out scientific research to explore such opportunities through national and international collaborative projects. However, the impact of RWE on routine signal management has been limited and concentrated in the later phases of signal management, by the largest and most well‐resourced organizations.

The aim of this article is to present recommendations to the pharmacovigilance, pharmacoepidemiology, and RWD partner communities regarding efforts that may enable broader and more robust use of RWD to generate RWE to support and enhance pharmacovigilance signal management. The process used to arrive at these recommendations is detailed in Appendix 1. These recommendations include changes to enable access to relevant RWD sources, timely development of RWE, and adaptations of processes and infrastructure, but do not cover the general conduct of pharmacoepidemiological analyses.

## 2 Concepts and Definitions

We recognize that the pharmacovigilance and pharmacoepidemiology communities have domain‐specific vocabularies to articulate their practice, sometimes using similar terms to represent different intents (e.g., confounding). Here, we define a set of concepts for the purposes of this paper and suggest that a prerequisite for further collaboration between pharmacovigilance and pharmacoepidemiology practitioners is ensuring a shared understanding of terms to ensure alignment of activities.

Real‐world data (RWD): RWD is defined as routinely collected data relating to a patient's health status or the delivery of health care from a variety of sources other than traditional clinical trials [13]. While individual case reports also describe real‐world experience and use, they are not included when we refer to RWD below. Similarly, we do not here focus on emerging sources of RWD such as patient‐collected data from wearable devices or patient‐generated data on the internet.

Real‐world evidence (RWE): RWE is the evidence derived from the analysis of RWD [13].

## 3 Signal Management

The CIOMS VIII working group on Practical aspects of signal detection in pharmacovigilance defines a drug safety signal as [1]:

Information that arises from one or multiple sources (including observations and experiments), which suggests a new potentially causal association, or a new aspect of a known association, between an intervention and an event or set of related events, either adverse or beneficial, that is judged to be of sufficient likelihood to justify verificatory action.

**FIGURE 1:**
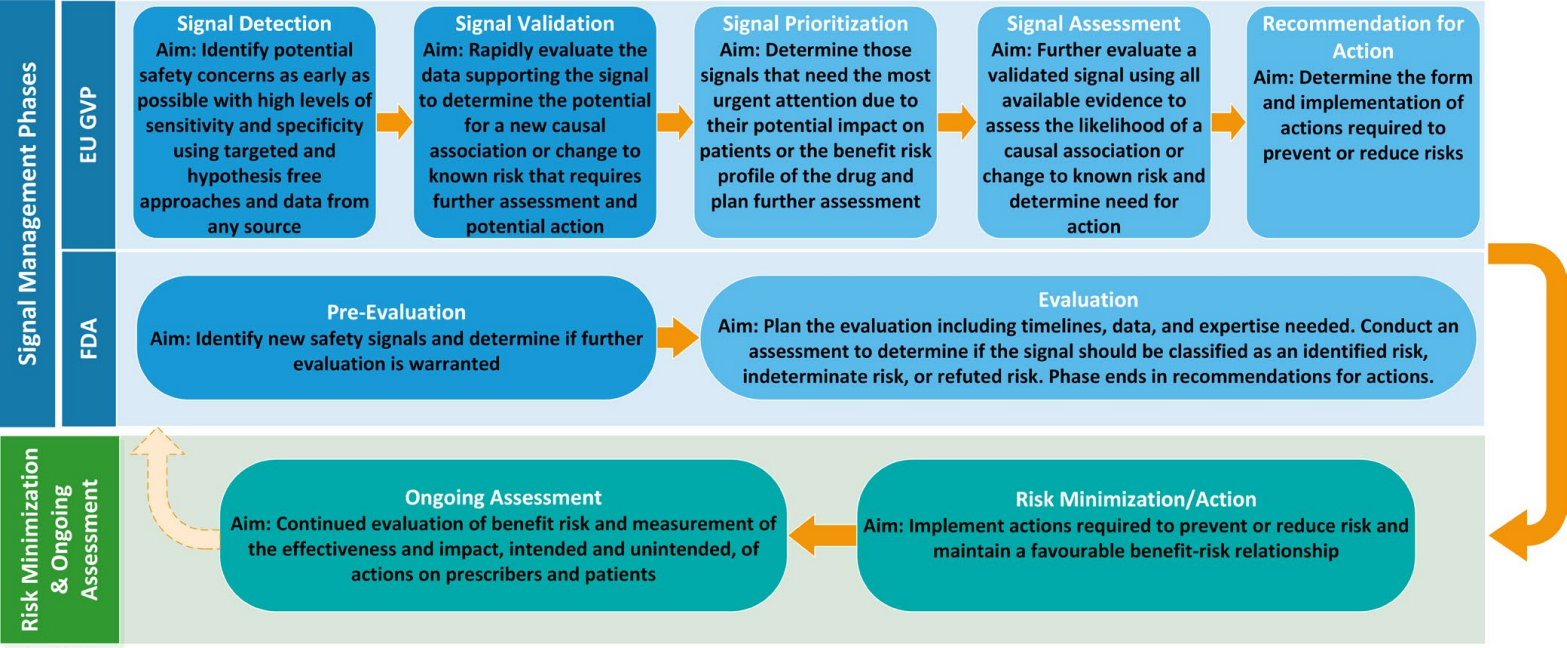
Pharmacovigilance signal management process outline. Different terms are used within different jurisdictions and organizations, and their precise delineations vary. N.B. Risk minimization and ongoing assessment are not considered phases of signal management but included here for context.

Since the study of beneficial effects in RWD presents additional challenges, and pharmacovigilance signal management typically targets adverse effects, this paper has the same scope. Figure 1 presents an outlined signal management process adopted by many organizations based on the European Union's Good Pharmacovigilance Practice guidelines and illustrates parallels to FDA's process for managing newly identified safety signals [14, 15]. Different terms may be used to describe the respective phases within different jurisdictions or organizations, and the precise extent of the analyses and type of evidence considered in each phase varies even within Europe [16]. Generally, the initial scope of signal detection is broad, covering a wide range of adverse events (and medicinal products, depending on the organization), reflecting the important requirement that pharmacovigilance be able to recognize and signal unexpected adverse effects. Progressing through the phases of signal management, the scope narrows as signals are closed, with more time and effort spent on each open signal in the later phases.

### 3.1 Signal Detection

Signal detection aims to identify information suggestive of potential unknown adverse effects as early as possible in the product life cycle. While signals can arise from various data sources, they often originate from human review of individual incoming case reports (sometimes guided by computational methods or heuristics [17]) or from statistical analysis of adverse event reporting patterns [18]. Some organizations apply expert judgment before considering a signal for further evaluation, whereas other organizations do this as part of their signal validation. While signal detection may include targeted monitoring of prespecified adverse events of special interest or importance, it must also be broad enough in scope to detect unanticipated adverse effects of almost any kind. Consequently, analytical methods must be computationally efficient and scale well to a range of different drugs and adverse events. As such, these analyses often have limited customization. A priority for this phase of signal management is reducing the probability that important signals are missed or delayed, while limiting the generation of false alerts.

### 3.2 Signal Validation

Signal validation forms part of an initial review which considers whether the signal relates to an already known adverse effect or may suggest a change in the magnitude of risk, is clinically relevant, and whether analyses are subject to potential biases such as notoriety bias, or if individual reports provide plausible alternative explanations (confounding factors such as underlying disease, co‐medications, etc.) or data quality problems like missing data, apparent errors, or case report duplication. Signal validation is usually performed by an expert evaluator and typically involves an analysis of the data that generated the signal—for example, review of individual case reports. It also considers available regulatory information, like product labels, and the scientific literature. While the balance between the speed and extent of the validation will vary between organizations and signals, its purpose is not to reach a final decision with regard to causality but to avoid attention being directed toward signals that are non‐actionable or for which the underlying data clearly does not suggest a causal association. As such, this phase should not require significant time or resources.

### 3.3 Signal Prioritization

Signal prioritization informs resource allocation within organizations, using all the information gathered in prior phases, to elevate signals most deserving of further investigation. This phase should also define the scope and timelines for the subsequent signal assessment. Criteria for prioritizing signals may include the novelty and strength of evidence associated with the signal and may consider the potential public health impact, such as the prevalence of use of the medicine in the population, the estimated rate, severity, and preventability of the suspected adverse reaction, and the vulnerability of the exposed population [19, 20].

### 3.4 Signal Assessment

Signal assessment involves the systematic and comprehensive analysis of all the evidence relevant to signals with the aim of assessing causality and better defining the potential impact on public health and individual patient safety. In this phase, different stakeholder groups may be involved, independent of who detected the signal. The evidence may derive from case reports, RWD, post‐authorization safety studies, clinical trials, and the scientific literature. Different criteria are used in the assessment of causality within pharmacovigilance, broadly based on the nine viewpoints proposed by Bradford Hill [21]. Further RWD sources are sometimes obtained, with extended timelines allowing for the development and evaluation of RWE. In this phase of signal management, a balance must be struck between timely but robust decision‐making with regard to taking actions to support patient safety and the scale of the evidence base used to support those decisions. When there is evidence to support a causal association, there may also be further clinical evaluation of the benefit‐risk balance, further investigation to characterize the risk in subpopulations, and evaluation of the impact of the occurrence of the event on the population (e.g., health system burden) and on an individual patient (e.g., quality of life, financial impact, personal health burden).

## 4 Use of Real‐World Evidence in Pharmacovigilance Signal Management

Three main uses of RWE in signal management are considered here: signal detection in RWD, provision of contextual information for identified signals, and analyses related to possible causal effects. We note that while action following signal evaluation, such as risk minimization measures including risk communication to patients and healthcare professionals and risk management evaluation, can also be a potential use case for RWE, it is out of scope for this paper.

### 4.1 Signal Detection

It has been hypothesized that RWD may complement individual case reports in signal detection, especially for multifactorial adverse events with high background rates in the population, where the attribution to a drug exposure is particularly difficult (e.g., myocardial infarction) [5, 22]. A possible benefit of RWD compared with individual case reports in signal detection is that it does not rely on adverse events having been recognized as possible adverse reactions and reported as such by patients or clinicians; on the other hand, this may increase the risk of spurious signals unless effective countermeasures can be implemented.

#### 4.1.1 Prior Research

A variety of methods have been proposed for hypothesis‐free signal detection in RWD. Recent reviews identify a significant body of research from 2005 to date [23, 24]. They note that comparisons between methods and approaches for signal detection are difficult due to the lack of harmonized terminology to describe analytical methods. There exist reference sets of corroborated adverse reactions [25, 26, 27] against which methods and datasets can be benchmarked, including for special populations [28]. However, only a minority of these include time‐indexed emerging safety signals [29], and favorable results in retrospective validation against well‐established adverse reactions may not generalize to routine signal detection performance, since the adverse event reporting and recording patterns differ between established adverse reactions and emerging safety signals [30]. In general, long‐term public access to reference sets developed as part of time‐bound scientific collaborations can be difficult to secure.

Multiple national and international collaborations have sought to implement and evaluate methods for safety signal detection in routinely collected health data. The Vaccine Safety Datalink is a US‐based collaboration in operation since 1990, which published some of the earliest research on signal detection using RWD, based on health insurance claims [31]. EU‐ADR was a European collaborative project that evaluated a range of methods for signal detection across a network of European datasets [32] and made comparisons between these data and spontaneous reports for signal detection [27]; one of its findings was that signal detection in a network of electronic health records data sources had a higher false positive rate than signal detection in a collection of individual case reports [5]. The US FDA Sentinel System was launched in 2016 following research and development in the Mini‐Sentinel pilot study since 2009 [33]; it originally focused on developing tools to support effective rapid‐cycle assessment of signals identified through traditional means but has in recent years expanded its capabilities for signal detection in RWD through a series of methods and development projects [34, 35]. In 2018, the FDA convened a public workshop to discuss methodological approaches and opportunities, as well as challenges involved with operationalizing signal detection capabilities in the Sentinel System [9, 36]. The Observational Medical Outcomes Partnership, OMOP, was a US‐based collaborative study with international partners that performed two rounds of major systematic evaluation of different methods for signal detection across different databases in the United States [37, 38]. IMI PROTECT was a European collaborative project that among other topics evaluated the use of UK primary care records for signal detection and validation [39]. Studies of signal detection in multicountry networks of RWD have also been carried out in the Asia‐Pacific region [40]. Observational Health and Data Sciences Initiative (OHDSI) is an international multi‐stakeholder collaborative seeking value from health data through large‐scale analytics. It provides several open‐source methods and tools aiming to support evidence generation, including signal detection, from RWD [11, 41].

#### 4.1.2 Applications

In view of the extensive research into pharmacovigilance signal detection based on RWD referred to above, there are surprisingly few records in the public domain describing examples where pharmacovigilance organizations have applied such methods as part of their routine drug surveillance operations and have been able to detect signals that have led to regulatory action. A recent scoping review included over 2400 pharmacovigilance signals between 1986 and 2019 [42]. Only 2.9% of these were based on epidemiological data and analysis, and most of these used epidemiological analyses to assess signals originally identified in other data sources, including clinical trials and individual case reports (D Sartori, personal communication, Apr 2024). Some pilot studies of signal detection in RWD have been performed. FDA recently completed three signal identification studies in the Sentinel System. While no statistical alerts were triaged as new signals in these analyses, the alerting patterns were consistent with known safety profiles for the products (i.e., semaglutide, erenumab‐aooe, and filgrastim‐sndz) [43, 44, 45]. Similarly, a pilot study within the IMI PROTECT project reviewed around 500 statistical signals from the THIN database of UK primary care records but closed more than 400 of these during signal validation; a small subset of the validated signals was selected for signal assessment, which identified cases of delayed diagnoses and contraindicated prescriptions [46] but no new adverse reactions. In France, researchers performed a pilot study of signal detection for noninsulin glucose‐lowering drugs with sequence symmetry analysis [47] and in Australia, the Therapeutic Goods Administration carried out a pilot study together with academic researchers exploring whether prescription sequence symmetry analysis could enhance signal detection using heart failure as a case study [48]. For vaccines, the FDA's Biologics Effectiveness and Safety (BEST) System and the Vaccine Safety Datalink have been used to conduct near real‐time surveillance for prespecified health outcomes [49, 50]. Untargeted signal detection methods have also been utilized for monitoring COVID‐19 vaccine safety [51, 52, 53].

### 4.2 Contextualization

An important task throughout the different phases of signal management is the contextualization of identified signals. This may consider drug utilization patterns and the characteristics of patients with the indication for treatment and/or the adverse event of interest. Today, such contextualization is often based on expert judgment, possibly supported by analyses of individual case reports, although these are subject to known selection biases. Analyses of RWD, on the other hand, could provide background incidence rates of a broad range of adverse events in those with and without exposure to medicines and thereby enable empirical identification or assessment of possible confounders and risk factors. Estimates of drug utilization and adverse event rates in RWD could also help determine the public health and patient impact of the potential risk.

#### 4.2.1 Prior Research

There is limited published research on how contextual evidence can be derived from RWD within the time frames of signal management. Isolated studies using reproducible analytical workflows have retrospectively reviewed the feasibility and value of descriptive analyses of UK electronic healthcare record databases to validate signals arising from individual case reports [54, 55]. A recent study has also demonstrated the feasibility of targeted search for additional cases in RWD sources for signals identified based on individual case reports [56]. A process for validating signals identified in RWD has also been proposed and evaluated [39]. More recently, a study including multiple European data sources in the European Health Data and Evidence Network (EHDEN) demonstrated the feasibility and potential value of rapid analyses of RWD within a federated data network to support decision‐making in signal validation and prioritization [57]. There has also been a pilot study of signal detection and prioritization for noninsulin glucose‐lowering drugs in a French healthcare database [47]. These studies have demonstrated that access to timely, on‐demand descriptive analyses of RWD can help reveal potential confounders and determine the feasibility and approach of subsequent epidemiological analyses. They have also highlighted the need for careful phenotyping of adverse events including mappings to common pharmacovigilance terminologies like MedDRA, and that sufficient breadth and depth of the RWD source are required to support use across a broader selection of the signals that may arise.

#### 4.2.2 Applications

In vaccine vigilance, the number of individual case reports of an adverse event with a specific medicinal product is sometimes placed in the context of the size and characteristics of the vaccinated population and the adverse event background rate in one or more RWD sources [58, 59]. However, studies have shown that there may exist substantial heterogeneity between data sources, which needs to be accounted for [60, 61, 62]. Descriptive analyses provide contextualization and are the most used modules during rapid‐cycle signal assessment in the US FDA Sentinel Initiative system [35]. Also, in the Data Analysis and Real World Interrogation Network (DARWIN EU, https://darwin‐eu.org) initiative of the European Medicines Agency, a large number of descriptive studies will be executed to provide context; DARWIN EU delivers RWE from across Europe on diseases, populations, and the uses and performance of medicines. Similarly, there is some use of the Clinical Practice Research Datalink for contextualization during signal validation at the MHRA in the United Kingdom (K. Donegan, personal communication, October 2023). A specific example of a multinational analysis within the OHDSI collaboration computed baseline incidence rates for adverse events of special interest in patients with COVID‐19 across 26 databases [63]. The validation and refinement of a new signal for colitis with nintedanib in individual case reports and RWD have been published as a proof‐of‐concept [64]. For signal prioritization, the more formalized approaches do consider external information sources and thereby offer opportunities for the use of RWD, but do not specify how the required RWE could be generated [19, 20]. There has also been a pilot study of what is referred to as the Longitudinal‐SNIP (strength, novelty, potential impact, and pattern of drug use) signal prioritization algorithm in France [47].

### 4.3 Analyses Related to Causal Effects

Signals will arise from real causal relationships but can also reflect random variability or bias (including confounding, measurement error, and selection bias). The execution of fully adjusted pharmacoepidemiological analyses in RWD can strengthen the evidence base for expert judgment, which may in turn promote consistency and transparency of decision‐making during signal management. The most important barriers to broader use of RWE for this purpose have been a lack of relevant RWD sources and the longer time typically required to access RWD and develop RWE than is available during signal management—especially in its earlier phases.

#### 4.3.1 Prior Research

Several national and international research initiatives have developed analytical infrastructure and methods to enable rapid‐cycle analyses capable of developing RWE within the time frames of pharmacovigilance signal assessment, including the aforementioned Vaccine Safety Data Link, FDA Sentinel Initiative, EHDEN, DARWIN‐EU, and OHDSI, as well as the Canadian Network for Observational Drug Effect Studies (CNODES) [65]. They have also established database networks that facilitate data access and enable simultaneous analyses of multiple databases to assess consistency and achieve statistical power for rare adverse events or drugs [8, 11, 12]. Key achievements include the development and deployment of Common Data Models for RWD sources providing harmonized data structures and representations [33, 66], of reproducible analytical workflows for faster design and execution of pharmacoepidemiological analyses in RWD sources, and, more recently, of standard phenotype libraries for relevant outcomes and covariates [67]. More‐EUROPA is an ongoing European collaboration aiming for more efficient use of RWD in the development, registration, and assessment of medicinal products.^1^

#### 4.3.2 Applications

There are several examples of the use of rapid‐cycle epidemiological analyses of RWD to analyze causal effects in support of signal assessment. However, they often require an extension of the normal regulatory timelines. Below, we outline two, for illustration.

After identifying compelling post‐marketing individual case reports of abnormal uterine bleeding in women of reproductive age on oral anticoagulants—an underrepresented demographic in clinical trials—US FDA evaluated the signal in RWD [68, 69]. These evaluations showed a higher incidence of severe abnormal bleeding in oral anticoagulant users compared to unexposed women, and differences in the risk across anticoagulant therapies. This information, combined with post‐marketing individual case reports, supported the addition of information on the risk of clinically significant uterine bleeding, potentially requiring gynecological surgical interventions, to the prescribing information of direct oral anticoagulants.

In the safety monitoring of COVID‐19 vaccines, a signal of myocarditis and pericarditis in young men, particularly following a second dose of an mRNA vaccine, was first detected based on individual case reports in Israel and the United States [70, 71]. These reports were contextualized in observed versus expected analyses, while preplanned rapid‐cycle analyses of multiple RWD sources in the United States, including through the Vaccine Safety Datalink and BEST Initiative, supported the suspicion of a causal effect and enabled rapid quantification of the outcome in temporal association with vaccination [72]. Subsequently, several population‐based cohort studies using RWD, including from the United States, United Kingdom, Scandinavia, Italy, and Korea, corroborated the finding and had a direct and rapid impact on global vaccination programs [73, 74, 75].

A survey by TransCelerate BioPharma indicates that 89% of the participating member companies currently do use RWD in signal assessment, with 44% using RWE for signal detection and 39% in signal validation. Half of the survey respondents specified 2 weeks or less as the required time frame to deliver rapid RWD analysis for signal assessment to fulfill company needs [76]. The most frequently reported challenge in the survey, identified by 8/18 (44%) companies, was the time required for analysis execution. Other challenges included availability or access to relevant RWD sources, uncertainty around the acceptance of non‐protocolized or minimally protocolized approaches by health authorities establishing relevant phenotypes or code lists, establishing a protocol, and the time required to access RWD data sources.

## 5 Inventory and Summary of Regulatory Guidance

A search of regulatory guidances was performed in Clarivate Analytics Cortellis for Regulatory Intelligence (CRI) database (Cortellis Competitive Intelligence and DTSR – Clarivate) to understand existing recommendations by regulatory authorities on the use of RWD and epidemiological methods to generate evidence across the pharmacovigilance signal management process. Overall, 63 regulatory guidance documents were identified. Many of these come from the United States Food and Drug Administration (US FDA) or the European Medicines Agency (EMA). Other countries with pharmacovigilance regulations often issue guidance documents with a similar structure and contents to the EMA pharmacovigilance guidelines (e.g., Middle East countries and Australia). For the most part, guidelines discuss signal management process regulations and provide information on signal detection from different data sources, investigation on whether signals are valid, and what criteria to use for signal prioritization. We did not identify in these guidelines explicit recommendations or requirements regarding the development of RWE in support of signal management. Several recommend that published RWE available in the scientific literature be considered as a relevant source of information throughout signal management. There is also general guidance on the analysis of RWD to support regulatory decision‐making, from, for example, the US FDA [77, 78] and publications from regulatory authorities that share experiences of integrating RWE in regulatory decision‐making, which include examples of rapid‐cycle analyses of RWD to support signal assessment [79].

## 6 General Obstacles to Broader Use of RWE in Signal Management

One common reason that RWE cannot inform signal management is the lack of relevant RWD sources, or their inadequate power, for a specific signal or setting. Unlike collections of individual case reports, each RWD source typically covers only a subset of adverse events (e.g., those that can be inferred by outpatient diagnoses or hospital admissions) and medicines of interest (e.g., those that are funded under an insurance plan or prescribed in a certain type of care). In some settings, linkage between sources may help overcome these challenges. However, in other settings, such infrastructure is lacking. While some RWD sources cover large populations, they may still not be sufficiently large to detect signals related to very rare adverse events or treatments. Research networks attempt to overcome this by coordinating systematic analyses across multiple datasets, including from different countries. However, for many parts of the world, reliable RWD sources are still lacking. Some RWD sources suffer from poor completeness, accuracy, or timeliness, which reduces their relevance and value for pharmacovigilance signal management.

A rate limiter when relevant RWD sources are available is the requirement for data access approval. It may be necessary to prespecify each drug‐outcome pair of interest, which is not feasible for signal detection and validation—especially in view of the considerable time often required for data access requests to be approved. For network studies, variations in data access requirements between RWD sources present additional obstacles and may slow down study execution.

Beyond that, the time required to design, execute, and analyze bespoke pharmacoepidemiological analyses is not always compatible with the urgency of pharmacovigilance signal management. Most of the analyses of RWD relevant to pharmacovigilance can be described as standard pharmacoepidemiological analyses, although the degree to which they can be customized to specific medicines and adverse events may be limited in the early phases of signal management. Clearly outlining the analytical designs and their customizable parameters and settings may speed up the execution of studies and improve their transparency and reproducibility [80]. Some methods for signal detection in RWD were originally adapted from other fields, such as pharmacovigilance, disease surveillance, and machine learning, which have resulted in a diverse and heterogeneous terminology that risks obscuring the similarities and differences between approaches. To ensure efficiency, transparency, and reproducibility, standardization of key components of the analytical workflows may be pursued. Reproducible analytical workflows provide structured and transparent data processing and analysis with clearly specified configurable parameters—not one‐size‐fits‐all. Use of publicly available analytical workflows eliminates redundancies and may facilitate quality control and assurance. Possible disadvantages of standardization are a lack of flexibility/ability to fully customize analyses, along with a greater initial cost of development when generalizability must be ensured. Further method development may be required to detect and assess signals in patient subpopulations, including pregnant women [81, 82] or those with specific conditions [35], as well as adverse reactions related to drug–drug interactions.

Access to multiple RWD sources may be necessary to enable detection and assessment of different types of signals and/or to achieve adequate power. The use of CDMs enables deployment of reproducible analytical workflows across multiple datasets and thereby the execution of network studies. Equally important may be the ability to rapidly execute analyses across suitable individual RWD sources depending on the signal of interest. A challenge introduced by using multiple RWD sources is the need to account for differences in health care systems and data capture processes when synthesizing and presenting analysis results. Disparate real‐world databases around the world use different source vocabularies (such as ICD‐9‐CM, ICD‐10, Read, ICPC), and there are varying approaches for how to achieve semantic harmonization when generating RWE. However, it is worth noting that further harmonization may be required for pharmacovigilance when synthesizing RWE with existing evidence that uses safety terminologies, including MedDRA.

The development and sharing of quality‐assured phenotypes for important outcomes and covariates has been identified as an important prerequisite for effective development and use of RWE [67]. In contrast, earlier research, for example, related to signal detection in RWD, often relied on individual medical terms or on standard groupings from the medical terminologies. Standard phenotypes for several outcomes and covariates have now been developed and made available by several of the larger collaborations [83], but more are needed to cover a substantial proportion of the relevant pharmacovigilance scope. Capability must also be developed to rapidly develop and validate phenotypes for emerging safety signals. To the extent that phenotypes can be implemented after mapping to a CDM, they can more rapidly be distributed and deployed across any datasets mapped to that CDM. However, if more specific adaptations are needed, customized phenotypes may need to be mapped from source codes within each RWD source and then mapped into the CDMs. The latter approach would reduce the risk of loss in translation but require more time and may be feasible perhaps only for the most prioritized outcomes and covariates.

A final obstacle to more extensive use of RWE to support decision‐making in pharmacovigilance signal management is the required changes to surrounding processes. Signal management relies extensively on human expert judgment, and the integration of new types of evidence will require guidance and best practices for how to critically appraise and combine this evidence with the evidence considered today. In addition, competence development or recruitment may be required to strengthen pharmacoepidemiological and RWD expertise within many pharmacovigilance organizations, and elsewhere, organizational changes may be needed to bring these competencies closer together. Pilot studies under realistic conditions may help define and drive this change, and sharing insights from such initiatives across organizations would help facilitate the transition.

## 7 Recommendations

### 7.1 Overarching

**Table:** 

Recommendation	Rationale
Foster cross‐disciplinary collaboration between pharmacovigilance specialists, pharmacoepidemiology specialists, and RWD partners, and strengthen multidisciplinary knowledge and perspectives.	Input from end users is vital to ensure that developed analytical methods and infrastructure are fit for purpose, meeting practical needs and requirements in pharmacovigilance. Effective integration of RWE in signal management requires synthesis of evidence from RWD, individual case reports, the scientific literature and more. This, in turn, requires collaboration between those with pharmacovigilance and pharmacoepidemiological expertise, as well as those with detailed understanding of the strengths and limitations of the analyzed RWD sources.
When possible, collaborate with other organizations to develop infrastructure, tools, and guidance for use of RWE in signal management	Many organizations face similar challenges. Pooling resources may help overcome the obstacles to broader use of RWE in signal management.
Develop and make available reference sets for benchmark evaluation of methods and RWD sources for diverse signal management use cases	Reference sets are required to understand operating characteristics of analytical methods and datasets. Benchmark evaluations should be possible for any stakeholder to execute for their environment. They can aid in interpreting evidence in light of expected false positive/false negative rates. They may also provide a basis for comparative evaluations of methods and RWD sources. For signal detection, they should include time‐indexed safety signals (with contextual information regarding which signals led to regulatory action, etc.).
Seek to ensure that resources created to support use of RWE in signal management (validated phenotypes, reproducible analytical workflows, benchmark reference sets, and more) remain available and open to continual updating and refinement based on feedback.	Pooling resources may help overcome the obstacles to broader use of RWE in signal management. Evolution of resources is necessary to reflect current scientific best practices and knowledge. Otherwise, they may not be relied upon for regulatory decision‐making. Whether a method meets required standards can ideally be empirically measured through benchmark studies.
To the extent possible, use standard pharmacoepidemiological terminology for describing analytical methods across the signal management use cases.	A common language for describing analytical methods will enable pharmacovigilance organizations to compare and choose between these. The descriptions should use standard epidemiological terminology when available and specify the individual analytical components and design choices to enable scientists to understand the unique and common aspects of different methods and identify opportunities for combining the strengths of different analytical strategies.

### 7.2 Pharmacovigilance Community

**Table:** 

Recommendation	Rationale
Be explicit about the value add of the use of RWE in each phase of signal management for your organization	The benefits of RWE in terms of incremental evidence supporting pharmacovigilance decision‐making must exceed the potential negative impact of unreliable RWE on decision‐making, and the opportunity cost of obtaining and analyzing RWE in lieu of synthesizing other evidence. Earlier signal detection may offset harm to patients, whereas elimination of false positive signals may benefit patient safety and public health and save resources for other activities etc. In general, more robust and comprehensive evidence may increase confidence in decisions.
Share experiences and insights from use of RWE in signal management with the broader community	Postmortem reviews in the scientific literature from both methodological and clinical perspectives are scarce but would enable the pharmacovigilance community to learn from each other's experiences. If scientific publication is not feasible, other modes of dissemination may be explored.
Determine the scope and nature of RWD required for prioritized use cases for your organization	The selected RWD sources should be able to answer a broad enough range of questions likely to emerge as part of the organization's signal management activities, so must include an appropriate set of medicinal products and adverse events and should cover relevant health care settings and appropriate geographies. Consider data catalogues such as https://catalogues.ema.europa.eu
Before incorporating analyses of RWE in signal management, ensure that your organization has the appropriate resources to do so effectively.	Current signal management activities are unlikely to be replaced in the short‐term (although there may be opportunities to close some signals more rapidly with timely access to RWE during signal management). Organizations with few resources, may await more solid tools/infrastructure and guidance, before starting to build this capacity.
If you plan to incorporate analyses of RWE in signal management, perform pilot evaluations for the integration of RWE in your pharmacovigilance use case(s) and share their results	Pilot evaluations enable assessment of performance under realistic conditions, help ensure that surrounding processes are well‐specified, and may allow organizations to evaluate operational considerations for effects on their current pharmacovigilance workflow.

### 7.3 Pharmacoepidemiology Community

**Table:** 

Recommendation	Rationale
Develop reproducible analytical workflows designed to support the pharmacovigilance signal management use cases, including when appropriate analytics for precomputed results; when possible, evaluate them against accepted benchmarks.	Pharmacovigilance operates under short timelines which require efficient analysis turnaround. Reproducible analytical workflows may generate reliable evidence in the required time frames. Analytics should be accompanied with objective diagnostics to verify that assumptions and limitations inherent to methods are evaluated and determined to be fit‐for‐use for the specific application. N.B.: Reproducible analytical workflows are not ‘one‐size‐fits‐all’, but rather consistent processes that, given a set of analytical design choices, will produce a defined set of analysis results. The analytical design choices, such as target, comparator, outcome, time‐at‐risk, outcome model, represent opportunities for customization within reproducible analytical workflows.
Develop, validate, and share phenotypes for important pharmacovigilance outcomes and covariates, including different levels of specificity and/or severity	Reliable RWE requires validated phenotypes for outcomes and covariates. Timely results require that these preexist for a wide range of medical events, and that there is capacity to rapidly develop and validate new phenotypes when needed.
Develop and maintain mappings between clinical concepts in MedDRA and standard vocabularies for RWD, for important adverse events	Pharmacovigilance signal management processes rely primarily on information encoded in MedDRA. Mappings from clinical concepts in MedDRA to phenotypes in the standard vocabularies for RWD (and vice versa) must be readily available and align on the clinical ideas that they seek to represent to enable rapid transitions between analyses in different types of data as well as evidence synthesis. Assumptions/limitations in developing such mappings should be acknowledged and transparently communicated.
To the extent possible, harmonize common data models (CDMs) for RWD	Efficiencies can be gained if we work with existing communities to evolve current standards rather than independently developing solutions de novo.

### 7.4 RWD Partners^2^

**Table:** 

Recommendation	Rationale
Develop and make available knowledge about your RWD source.	Accurate interpretation of RWE requires a detailed understanding of each RWD source. Ideally, a representative of each data partner may be part of the study team, but this is not always possible. In addition to overall descriptive statistics, meta‐data should be made available such as how information was captured, by whom, and in which populations, etc., as well as listing any known biases or exclusions. For examples of relevant meta‐data, see https://catalogues.ema.europa.eu
Adopt streamlined data access approval processes and harmonize requirements for study protocols across RWD sources.	Pharmacovigilance operates under short timelines and, depending on the signal, may need access to multiple sources of RWD to support signal management, either separately or in a network study. The greater the efforts and longer the waiting times to secure approvals, the less likely it is that RWD will influence decision‐making during signal management.
Consider data access approval for overarching study designs/master protocols for pharmacovigilance use cases.	When pharmacovigilance signals are detected, they need to be assessed with minimal delays. Pharmacovigilance is on the lookout for the unexpected and can generally not be restricted to a small set of drug‐outcome pairs of interest. Overarching study designs/master protocols may use prespecified (and benchmarked) reproducible analytical workflows but include broad sets of drug‐outcome pairs of potential interest.
Map datasets (and maintain mappings) to the broadly used Common Data Models, including their standard vocabularies	Up‐front mapping of data to a Common Data Model (or to multiple CDMs) including standard vocabularies enables execution of reproducible analytical workflows within the restricted time frames required to support pharmacovigilance signal management, and the inclusion of the RWD source in network studies.
Develop capability and infrastructure to execute reproducible analytical workflows in support of major signal management use cases	To enable timely generation of RWE in support of the signal management use cases.
Consider embedding data collection in support of pharmacovigilance and pharmacoepidemiological analyses within existing or new health data systems	Targeted collection of more detailed data would enable RWD analyses that can better support signal management and also execution of active surveillance/registry collection within existing health data systems. This may include capturing information on brand names, lack of efficacy or exacerbation of other conditions, indications for treatment, differential diagnoses, or explicitly confirmed treatment/outcome start dates to reduce the risk of misclassification.

## 8 Discussion

Substantial efforts over nearly 20 years have sought to develop and evaluate methods that can enable pharmacovigilance organizations to use RWD and generate RWE to support decision‐making in signal management. There are reasons to believe that this could enhance decision‐making throughout signal management and that signal detection in RWD may enable more effective identification of certain risks. However, with few exceptions, efforts to incorporate RWE have so far had limited impact on routine pharmacovigilance activities, with most organizations still relying largely on analyses of individual case reports combined with preexisting evidence obtained from regulatory information and published scientific articles—especially during signal detection and validation.

This paper highlights a range of opportunities to enable broader use of RWE in pharmacovigilance signal management and obstacles that must be overcome to integrate RWE more fully. These include lack of access to relevant RWD sources for a wide variety of signals and regions and technical limitations, such as a lack of validated phenotypes for a broad enough range of important adverse events and covariates and reference sets for benchmark evaluation of methods and RWD sources. However, strategic and operational obstacles may be equally important. Strategically, organizations need to decide whether (and when) to invest resources in capacity to obtain and integrate RWE in signal management. This may require that decision‐makers within the organization and those providing external funding (for example, ministries of health in the case of government organizations) also understand and appreciate the value of RWE for pharmacovigilance signal management. The absence of compelling use cases that illustrate the possible benefits (and costs) of doing so constitutes a potential dilemma, with weak incentives for pharmacovigilance organizations to invest in such capability which, in turn, would extend the community's experience and insights. Operationally, organizations deciding to integrate RWE in signal management will need to build infrastructure, competence, and supporting processes, which is complicated by the lack of validated best practices. For pharmaceutical companies, there is the additional obstacle that their pharmacovigilance processes must comply with regulatory requirements which are currently lacking in detail.

Ideally, the development of use cases, best practices, and regulatory guidance may be advanced and catalyzed by ongoing work of large regulatory authorities. As insights from initiatives like FDA Sentinel and DARWIN EU grow and are shared, a clearer view may emerge of when and how RWE can best support and enhance signal management. There may also be untapped resources of insights related to effective use of RWE during signal management: the TransCelerate BioPharma survey suggests relatively broad use of RWE during signal assessment within the pharmaceutical industry [76], and yet there are comparatively few publications that describe this. Other organizations may learn from and build upon such experience, but each stakeholder will need to secure their own ability to obtain and analyze RWE and adapt any insights regarding the optimal integration of RWE in signal management to their own setting. In this context, the lack of access to RWD sources in some parts of the world may reduce the relevance of RWE in signal management for their populations at present. On the other hand, if digital health systems in these countries can be developed from the ground up with the pharmacovigilance use case in mind to ensure fit‐for‐purpose and robust information capture, they may be better off in the long term. Some analyses of RWD in support of signal management are possible already today for organizations with pharmacoepidemiological expertise, analytical infrastructure, and access to RWD sources. They include rapid‐cycle signal assessment and targeted signal detection restricted to specific, well‐defined, and captured adverse events and medicines, for which there are validated phenotypes.

A prominent concern has been that signal detection in RWD may generate large numbers of false alerts, and that such signals may be difficult to assess if the RWD sources best suited for epidemiological evaluation have already been used during signal detection, noting that subsequent analyses in the same RWD source may be valid only under certain circumstances [84, 85]. In this context, the dearth of signals detected in RWD to date suggests that false negatives (missed signals) may be an equally important, arguably under‐appreciated, challenge.

A strength of this project is that it brought together experts from regulatory authorities, pharmaceutical companies, academia, and international organizations. It received support from ISPE and performed a formal consultation of the final manuscript open to all members of this society. As a complement, during its development, members of the ISoP Real‐World Evidence Special Interest Group and the OHDSI annual meeting in 2023 were invited to provide feedback on the draft recommendations. The project did not perform a systematic review of prior research or current use of RWE in signal management but consulted two recently published review articles and performed an inventory of regulatory guidance documents in this area.

This paper calls for efforts by the pharmacovigilance, pharmacoepidemiology, and RWD partner communities to enable broader and better use of RWE in pharmacovigilance signal management in the future. As the obstacles outlined here are overcome and the body of evidence regarding effective use of RWE for signal management expands, practical guidelines/frameworks for use of RWE in pharmacovigilance signal management should be developed.

### 8.1 Plain Language Summary

Before medicines and vaccines are approved for general use, their safety and effectiveness must be demonstrated. Even after approval, they must be continually monitored by regulators, pharmaceutical companies, and others for new information on adverse effects that may change how the balance between their benefits and risks is understood. The science and activities related to this are referred to as pharmacovigilance. Since the 1960s, it has relied extensively on the collection and analysis of individual case reports, which have well‐known strengths and weaknesses. More recently, there have been efforts to use routinely collected health data to rapidly generate more robust epidemiological evidence as a complement. However, this research has had limited impact on routine pharmacovigilance so far. This paper offers recommendations to support broader use of real‐world evidence in this field. These include streamlined data access and reproducible analytical approaches that work across different data sources. The authors stress the importance of cross‐disciplinary collaboration, organizational adaptations, and pilot studies under real‐world conditions to help move the field forward.

## Disclosure

The views expressed are those of the authors and do not necessarily represent the position of, nor imply endorsement from, their affiliated regulatory agencies or organizations.

## Conflicts of Interest

G. C. is employee of Bayer AG. AB is employee and shareholder of GSK. MSG is former employee of Bayer AG and shareholder of GSK and Bayer AG. PBR is employee and shareholder of Johnson & Johnson. G. T. has served, over the last 3 years, on advisory boards/seminars funded by Sanofi, MSD, Eli Lilly, Sobi, Celgene, Daiichi Sankyo, Novo Nordisk, Gilead, and Amgen on topics not related to content of this paper; he is also a scientific coordinator of the academic spin‐off “INSPIRE srl,” which has received funding from several pharmaceutical companies (Kiowa Kirin, Shionogi, Shire, Novo Nordisk, and Daiichi Sankyo) for conducting observational studies and additional consultancy services on topics not related to content of this paper. In addition, he is currently a consultant for Viatris in a legal case concerning an adverse reaction to sertraline. All other authors declare no conflicts of interest related to this work. This paper addresses general principles for integrating real‐world evidence into pharmacovigilance signal management and does not consider specific medicinal products.

## Endnotes

^1^

https://umcgresearch.org/en/more‐europa.

^2^
These recommendations apply to RWD partners (RWD source holders/owners/managers) interested in their data being used in support of pharmacovigilance signal management.

## Appendix 1

Process to Develop Recommendations.

The conception of this project originated from a proof‐of‐concept study integrating real‐world evidence (RWE) in pharmacovigilance signal validation and prioritization within the IMI EHDEN public–private partnership. A subset of the authors of this publication (NN, PR, PR) who participated in that study recognized the need to bring the pharmacovigilance and pharmacoepidemiology communities together further to increase the appropriate use of RWE through the medicinal product safety lifecycle.

The group developed a proposal under the International Society for Pharmacoepidemiology (ISPE) Funded Manuscript Program. As part of that process, a working group of pharmacovigilance and pharmacoepidemiology practitioners was brought together with an aim to be representative of the diverse stakeholders (including regulatory agencies, academia, and pharmaceutical industry), geographies (including United States, Europe, and Asia‐Pacific regions) and disciplinary perspectives (including medical informatics, statistics, epidemiology, health policy) that could contribute expertise to this project. The proposal was submitted in September 2022 and selected for funding by ISPE's Public Policy and Publications & Communications Committees later that year.

The workgroup convened to draft recommendations, meeting virtually on a monthly basis and working asynchronously through shared documents over the course of 8 months in 2023. Consultations on the draft recommendations were held with participants in the Observational Health and Data Sciences Initiative (OHDSI) Annual meeting on Oct 22, 2023, and with the International Society of Pharmacovigilance (ISoP) Special Interest Group on Real‐World Evidence and Big Data on Nov 29, 2023. Four leading specialists in the area (coauthors AB, ST, GT, and RP) were initially invited as dedicated reviewers for the draft manuscript. Following their contributions, they were invited to participate as coauthors and then contributed fully to the final stages of the manuscript development.

The final manuscript draft was subjected to a review open to all members of the ISPE, revised based on contributed feedback, and was subsequently endorsed by ISPE's Board of Directors.
